# Impact of pH and feeding system on black soldier fly (*Hermetia illucens*, L; Diptera: Stratiomyidae) larval development

**DOI:** 10.1371/journal.pone.0202591

**Published:** 2018-08-27

**Authors:** Marco Meneguz, Laura Gasco, Jeffery K. Tomberlin

**Affiliations:** 1 Department of Agricultural, Forestry and Food Sciences, University of Turin, Grugliasco, Turin, Italy; 2 Department of Entomology, Texas A&M University, College Station, Texas, United States of America; Universita degli Studi della Basilicata, ITALY

## Abstract

Black soldier fly (BSF) is a generalist species able to reduce large quantities of organic substrates and is thus considered as an interesting solution for waste management. Moreover, as BSF larvae accumulate high quantities of nutrients during their growth, they are valued because of their potential to produce products such as protein meal or fat for livestock feeds. Abiotic factors can influence larva growth, and a more detailed knowledge and control of these parameters can lead to the development of mass BSF breeding for the production of innovative products for animal feeds. As little information is available on the effects of the pH of substrates and feeding systems, the aim of this study was to evaluate the impact of these two factors on the activities of BSF larvae, prepupae, and adults. An experiment was performed with two fixed factors: i) pH (4.0; 6.1; 7.5; 9.5) and ii) feeding system (batch feeding system (TFS) or daily feeding system (DFS)). The pH treatments impacted larval weight on the first, third, and fifth day, but not at the end of the trial. Larval activity increased pH values from the fourth day onward, with final values of around 8.9–9.4 in all the treatments. The weight of the prepupae ranged from between 0.094 and 0.100 g. The final weight of the larvae and pupae, sex ratio, ingested food, larval mortality, percentage of emergence, and time to reach the pupa stadium were all affected by the feeding system. DFS showed the heaviest final larval weight (0.149 g), but required a longer time (11.3 d) than TFS to reach the prepupa stadium. The findings of this research could be useful for the mass production of BSF. Evaluation of an appropriate feeding system and initial pH value of the substrate are important parameters to reduce the time and to increase the weight in the production of larvae.

## Introduction

The book "Limits to Growth" [1], published more than 40 years ago, emphasized the expected exponential growth of the human population would be in conflict with limited land and other resources available to support it. These issues have become a reality: the Food and Agricultural Organization of the United Nations (FAO) estimated the world population was 7.2 billion in 2014 with 2 billion being added from 1990–2014. Estimates on this continued growth indicate the human population will reach 9 billion by 2050 [2]. The daily energy intake per individual is also expected to increase from 2770 kcal presently to 3070kcal by 2050; these factors together suggest an increase in food production will be needed [3] for both food and feed uses, and, as a result, additional land will be needed to produce several key commodities (i.e. cereals, protein-containing foods). These requirements will place pressure on land and renewable water and increase environmental changes [3]. An equal redistribution of produced food will not be sufficient to close the food gap. The FAO’s “World Agriculture towards 2030/2050” report stated about 815 million people in the world were undernourished in 2016 [4], and this number will increase dramatically in the future. Therefore, new sources of food are necessary, especially as far as protein is concerned. This population growth and increased welfare will be responsible for increased meat consumption and production especially in developing countries. Forecasts show an increase of 2.7% per year of meat, which is higher than the 1.4% reported earlier [3].

The global production of feeds has reached 1.07 billion metric tons, and the trend is expected to continue to increase in developing countries [5]. As proteins are the most important component of animal feeds, new alternative sources which should not compete with land associated with soy or other crop production, are needed. Some innovative protein sources, such as algae [6], fungi [7,8], and insects have been investigated; the latter seems to be the most promising [9,10].

Insect farming is a viable solution, due to the low land and water requirements [11], and because insects can be fed pre- or post-consumer food waste [12,13]. Globally, more than 2000 insect species are consumed daily by humans, but only a few have been studied in detail to establish for their use as livestock or aquaculture feed. Some of the most studied species are the yellow mealworm (*Tenebrio molitor* L. (Coleoptera: Tenebrionidae)) [14], black soldier fly (BSF) (*Hermetia illucens* L. (Diptera: Stratiomyidae)) [15,16,17] and house fly (*Musca domestica* L. (Diptera: Muscidae)) [18].

BSF colonizes numerous types of decaying organic matter, including animal manure, fish offal, food waste, and vegetable waste [19,20]. This species is distributed throughout the tropics and temperate regions of the world [21]. Over the past 30 years, BSF has been studied because of its potential to reduce large quantities of organic waste. The mass production of BSF can result in several products such as protein meal [22,23,24,25,26] and fat [27,28] for feed purposes or for biodiesel production [13] as well as fertilizers [29]. As in any livestock production, abiotic factors can also influence the growth in BSF rearing, and the correct knowledge and control of these parameters can lead to the development of methods for optimizing BSF production.

The effects of substrate quality on the growth of BSF larvae have already been studied. In many cases, these studies explored the impact of broad categories of substrates on BSF development [12,15,20,30,31], survivorship [16,32,33], and conversion rates [13,30,34,35]. The role of temperature and humidity on the life-history of BSF larvae was evaluated by Holmes et al. [33], who found these two factors can impact larval development, mortality, and the emergence time of adults.

An intrinsic parameter in substrates having been examined in part is pH. Popa and Green [36] evaluated the effects of pH in a liquid medium (leachate) on larval development. Their results demonstrated larvae were able to regulate the pH to a value of almost 9.0. Allatar [37] determined BSF larvae had an impact on the pH of a liquid medium, but also indicated this impact was dependent on larval density (0.005 g/ml). Like Popa and Green [36], the author determined pH of the medium ranged between 8 and 9. So far, to the best of our knowledge, only one study has investigated the role of the initial pH on the development of BSF larvae. Ma et al. [38] investigated the effect of different initial pHs (2.0, 4.0, 6.0, 7.0, 8.0 and 10.0) on the growth performances of BSF larvae. The study pointed out how pH values of 6.0 to 10.0resulted in heavier final BSF pupal weights than lower pH values (2.0 and 4.0) and pointed out larvae regulated the final pH values from 8.0 to 8.5, except for 2.0 and 4.0, which showed a final pH value of 6.0.

Other studies have evaluated the effect of pH on other dipterans. Diets for the primary screwworm *Cochliomyia hominivorax* (Coquerel) (Diptera: Calliphoridae) with a pH close to 7.5 produced larger pupae than those reared on a diet with pH close to 6.8; those reared on a more acidic pH (4.0) produced the smallest pupae [39]. The growth of larval Mediterranean fruit flies, *Ceratitis capitata* (Wiedemann) (Diptera: Tephritidae) is optimal when reared on artificial diets with a pH of 5.0–5.5 [39, 40]. As little information is available on the effects of substrate pH and feeding systems, on the mass production of BSF, the aim of this study has been to evaluate the impact of these two factors on the larval and pupal development, as well as mortality and adult emergence of BSF.

## Materials and methods

### Colony

BSF eggs were collected from a colony at the Forensic Laboratory for Investigative Entomological Sciences (FLIES) Facility at Texas A&M University, College Station, TX, USA. Eggs < 24-h-old were collected using the methods described in Sheppard et al. [41] and were stored in a Rheem Environmental Chamber at 27°C, at 70% relative humidity and with a 14:10 light-dark cycle until hatching. The neonates, consisting of a batch of more or less 45 thousand larvae) were fed with a Gainesville diet (~200g, from day 0 to day 5), which is composed by 30% alfalfa, 50% wheat bran and 20% corn meal [42], with a 70% moisture content.

### Experimental design

A completely randomized and fully crossed experiment was conducted considering two factors: i) 4 different pH levels (4.0; 6.1; 7.5; 9.5), and ii) 2 different feeding systems (batch feeding system (TFS) or a daily feeding system (DFS)). Four replicates were made per treatment.

### BSF rearing

Five day old larvae were used in the experiments. Larvae were partitioned into allotments of 500 larvae in 8000 cm^3^ plastic containers (33.5 x 22.8 x 10.9 cm; H-E-B, San Antonio, TX, USA). Cups containing larvae were then placed, in a randomized design, on shelves in a climate chamber in which all the environmental conditions were controlled (29 °C and 70% RH). A data logger (Hobo U12-006, Onset Computer Corporation 470 MacArthur Blvd. Bourne, MA, USA) was used to monitor the temperature and humidity. Ten larvae per replicate were randomly selected daily and weighed using an Adventure Pro Balanec (Ohaus, Pine Brook NJ, USA; d: 0.0001) and were then returned to their respective containers. The larval rearing trials were stopped when 40% of the larvae reached the prepupal stadium in each replicate. When a replicate was stopped, remaining larvae were cleaned, and weighed and total survival recorded.

Prepupae were placed in a plastic cup (33.5 x 22.8 x 10.9 cm; H-E-B, San Antonio, TX, USA) stored in the same climate chamber under the same conditions used previously described. When the prepupae became pupae, ten individuals from each replicate were weighed individually. The percentage of emergence and sex ratio of the entire cohort were recorded. In order to evaluate the differences in weight on a DM basis, adults were sexed and oven dried (55°C, Thelco laboratory 6540, Thermo Fisher Scientific Inc., Marietta OH, USA)) until the samples stopped decreasing in weight.

Remaining residues were weighed and dried (55°C, Thelco laboratory 6540, Thermo Fisher Scientific Inc., Marietta OH, USA). The remaining residues should have only consisted of frass, but could also have contained small amounts of non -digested substrates. In order to evaluate whether the pH and feeding system affected the consumption of larvae, the amount of digested diet was calculated, in percentage, on a dry matter basis (DM) using: Ingested food = (Provided food—remaining residues/ distributed food)*100.

### Substrates pH management

The Gainesville diet [42] was used as the substrate to perform the experiments, with a moisture content of 70%. Four different pH treatments were examined. Diet pH was manipulated using the following method. Citric monohydrate acid (Hearthmark, LLC dba Jarden Home Brands; Fishers, IN 46037, USA) (0.1 M) was added to the total amount of the diet, which was calculated on a daily feeding basis, as described in Tomberlin et al. [15], to decrease the pH to 4.0 ± 0.1. A sodium hydroxide (NaOH) (Sigma-Aldrich Co., Saint Louis, MO, USA) solution (0.1 M) was added to increase the pH to 7.5 ± 0.1 (low alkaline) and 9.5 ± 0.1 (high alkaline). The diet without any manipulations was used as the control (pH = 6.1 ± 0.1).

### Feeding systems

The amount of food provided during the trials was calculated on the basis of the feeding rate of the Gainesville diet (40 g/daily on wet basis (WB)). The total amount of the TFS diet was calculated according to the time the local colony took to reach the prepupa stadium (480g on WB) and was distributed at the beginning of the trial, and no further amount of diet was added during the trial. An aliquot of 40g (on WB) of diet was provided daily for DFS. The exact amount of food provided in each replicate was recorded and used to calculate the Ingested food parameter.

### pH evaluation

Daily, pH was measured in each replicate across treatments using by collecting 10 g substrate, placed in 50 ml glass beaker with 17 ml distilled water, and pH recorded (based on Chan and Jang [40]) using a pH meter (Hanna Instruments, H5221, Woonsocket, RI 02895; USA). Each sample was tested three times. After the measurement, the sample was reintegrated to its respective replicate. Moreover, sampling of the DFS replicates was performed before the new batch of food was provided.

### Statistical analysis

The statistical analysis of the data was performed with IBM SPSS statistic v 20.0 for Windows. The normality of the data was evaluated, larval mortality (%), time and percentage of emergence of adults (%) were transformed with a square root and determined to meet parametric analysis requirements. A repeated measure ANOVA accounting for two fixed effects, “pH treatment” and “feeding system”, and a repeated measure treatment, “time” expressed in days was applied on larval weights. Interactions between the two main factors (pH and feeding system), pH and time, the feeding system and time, and the overall interaction (pH, feeding system and time) were tested. Levene’s test was used to verify the homogeneity of variance for each combination of the groups of within- and between-subject factors. Mauchly's test was used to verify the assumption of sphericity; if such an assumption was violated, the Greenhouse-Geisser or the Huynh-Feldt correction was applied (in the cases where the sphericity estimates were lower or higher than 0.75, respectively) to correct the degrees of freedom of the F-distribution. Tukey’s post hoc test was used to evaluate the differences between the pairs. The final larval and pupal weight, the adult weight of the males and females (g on DM), larval mortality (%), the percentage emergence of adults (%), the time to reach the prepupae stadium and ingested food (% on DM) were analyzed by means of two-way ANOVA. Tukey’s post hoc test was used to evaluate the differences between the pairs.

## Results

Temperature and humidity recorded during the trial were 29.3 ± 1.4°C and 70.0 ± 5.0%, respectively. No treatment indicated adverse feeding and rearing conditions.

### BSF growth performance

**Fig 1:**
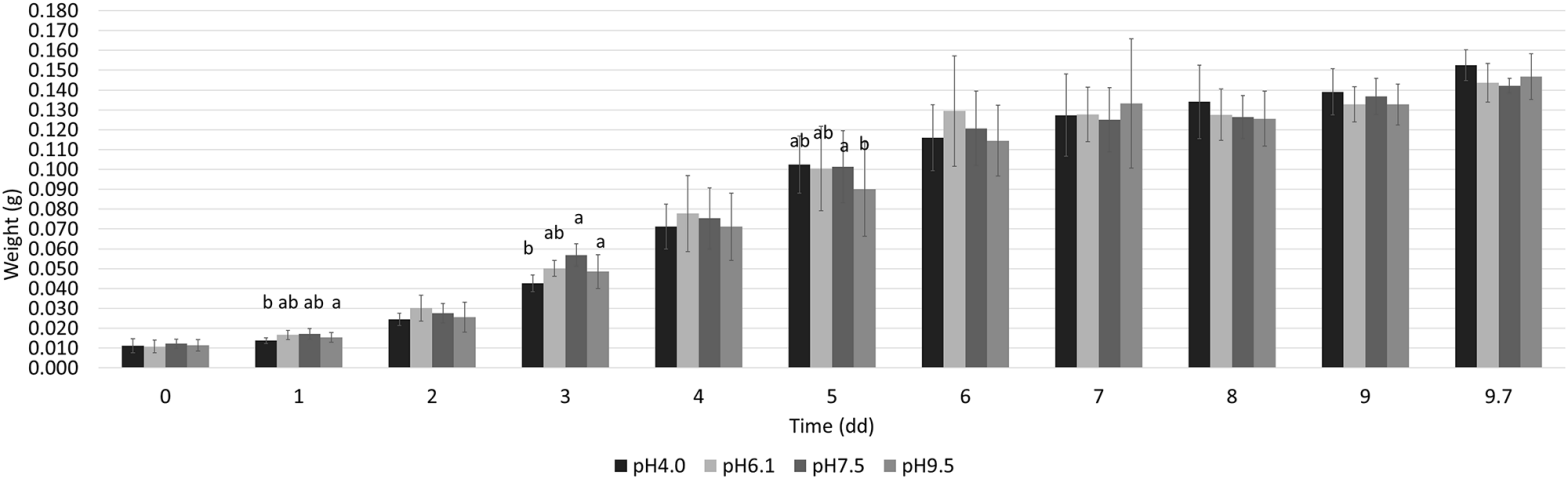
The average weight (means ± standard deviation (SD)) of BSF larvae reared on diets with different pH levels (pH4.0; pH6.1; pH 7.5; pH9.5) over time. Bars topped with different letters indicate a significant difference (*P*≤0.05) as inferred by post-hoc pairwise comparisons.

Initial larval weights (0.011 ± 0.0028 g) were not significantly different between treatments (P = 0.842). Diet pH impacted larval weight on day one, three, and five, but the larvae did not show any differences at the end of the trial (F3,24 = 1.98; P = 0.143), as shown in Fig 1. The final weight of the larvae reared on different pH diets ranged from between 0.142 and 0.153g.

The influence of pH on the larval weight over time is presented in Fig 1. An interaction between pH and time was recorded over the experiment (*P*≤0.05).

**Fig 2:**
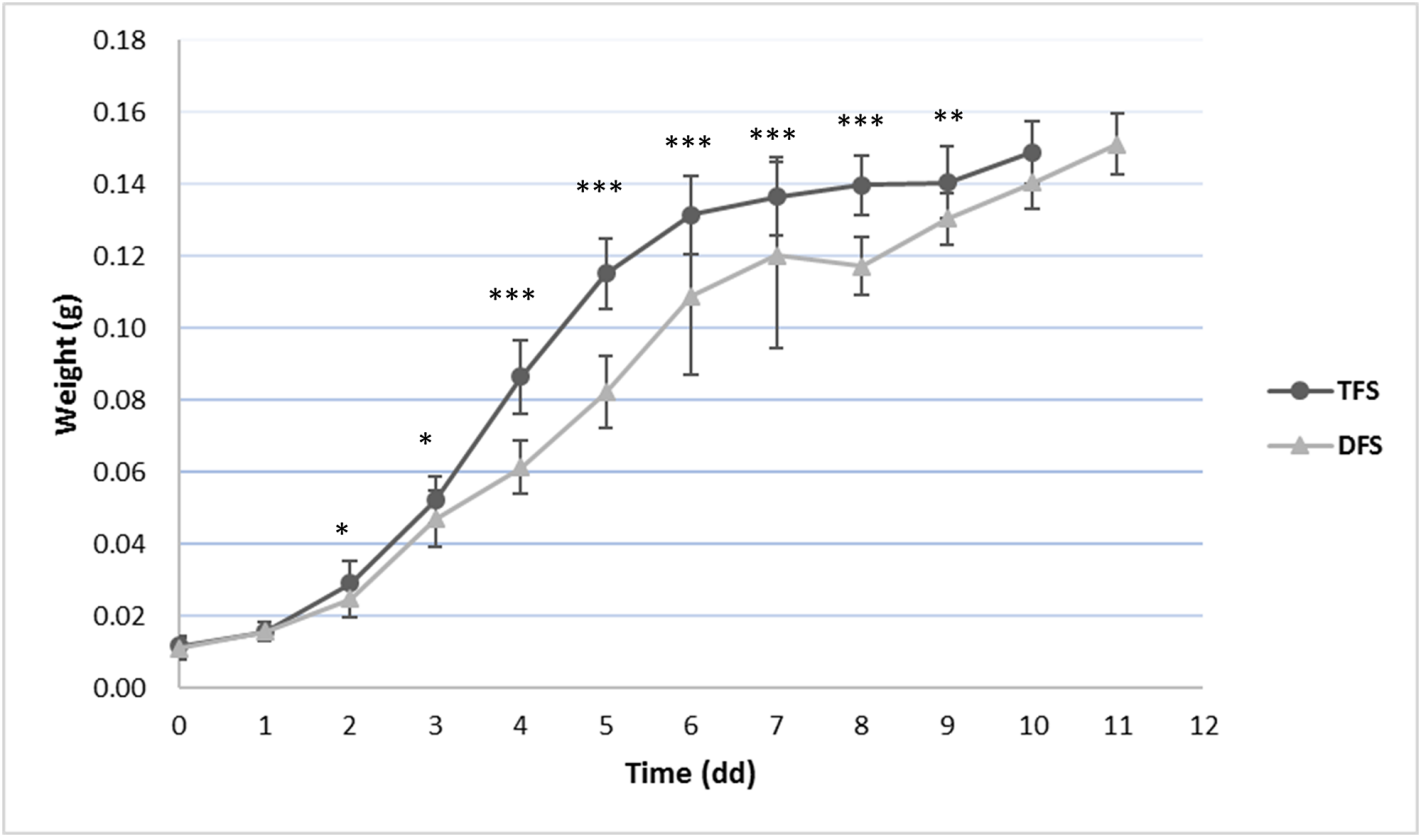
Average weight (mean ± SD) of the BSF larvae reared on two different feeding systems; TFS: Total amount of diet provided at the beginning (480g on WB); DFS: Daily allocated diet (40g/d on WB)). *: *P*≤0.05; **: *P*≤0.01; ***: *P*≤0.001.

The feeding system had a significant impact on the larval weight during days two to nine. The TSF treatment was ended on day 10, while a further day was needed for DFS to achieve 40% of the prepupa stadium (Fig 2). An interaction between the feeding system and time was recorded over the experiment (*P*≤0.05). No interaction was recorded between pH and the feeding system over the days of the trial (P = 0.455).

The overall interaction (pH, feeding system and time) did not show a significant level (P = 0.677).

The final larval and pupal weights (g), the adult male and female weights (g on DM), sex ratio, ingested food (% DM), percent mortality of the larvae (%), percent emergence (%), and the time to emergence (d) are shown in Table 1.

**Table 1:** Life history traits of the larvae and adults of BSF reared on diets with different pH (pH4.0; pH6.1; pH7.5; pH9.5) and with different feeding systems (TFS: Total amount of diet provided at the beginning (480g on WB); DFS: Daily allocated diet (40g/d on WB)). Mean ±SD. DM: dry matter. Different letters within a column (life history trait) indicate significant difference for *P*≤0.05.

	Final larval weight (g)	Pupal weight (g)	Male (g on DM)	Female (g on DM)	Sex ratio 1:1 (Male:female)	Ingested food (% DM)	Larval mortality (%)	Emergence (%)	Time (d)
pH4.0	0.153±0.0113	0.100±0.0096	0.012±0.0011	0.014±0.0012a	1.8±0.35	60.9±2.60	3.5±3.28	96.2±2.32a	10.7±0.55
pH6.1	0.144±0.0127	0.094±0.0120	0.012±0.0015	0.014±0.0021a	1.7±0.68	61.0±2.89	4.6±4.08	95.3±3.92a	10.5±0.95
pH7.5	0.142±0.0065	0.094±0.0160	0.011±0.0014	0.012±0.0011b	1.7±1.14	60.2±4.88	6.0±5.74	82.4±14.19b	10.3±1.16
pH9.5	0.147±0.0058	0.095±0.0012	0.011±0.0011	0.014±0.0014a	2.2±0.80	58.6±3.65	3.1±2.28	88.4±10.67ab	10.5±0.87
Test statistic	Df = 3,24; F = 1.98; *P* = 0.143	Df = 3,24; F = 0.88; *P* = 0.466	Df = 3,24; F = 2.70; *P* = 0.068	Df = 3,24; F = 3.40; *P* ≤0.0345	Df = 3,24; F = 1.43; *P* = 0.258	Df = 3,24; F = 0.00; *P* = 0.413	Df = 3,24; F = 0.162; *P* = 0.921	Df = 3,24; F = 7.25; *P* = 0.004	Df = 3,24; F = 10.12; *P* = 852
TFS	0.142±0.0105b	0.104±0.0061a	0.012±0.0004	0.013±0.0009	1.6±0.43b	58.2±1.32b	3.2±2.16b	85.6±5.80b	9.7±0.24b
DFS	0.149±0.0008a	0.088±0.003b	0.011±0.0013	0.013±0.0005	2.1±0.28a	62.1±1.45a	5.3±2.17a	95.6±2.82a	11.3±0.05a
Test statistic	Df = 1,24; F = 4.72; *P* = 0.040	Df = 1,24; F = 24.38; *P* = 0.000	Df = 1,24; F = 0.23; *P* = 0.637	Df = 1,24; F = 0.00; *P* = 0.975	Df = 1,24; F = 7.14; *P* = 0.013	Df = 1,24; F = 13.92; *P* = 0.001	Df = 1,24; F = 7.00; *P* = 0.021	Df = 1,24; F = 10.24; *P* = 0.001	Df = 1,24; F = 227.89; *P* = 0.000
Interaction factor	Df = 3, 24; F = 0.423; *P* = 0.738	Df = 3, 24; F = 0.170; *P* = 0.193	Df = 3, 24; F = 0.216; *P* = 0.884	Df = 3, 24; F = 0.306; P = 0.821	Df = 3, 24; F = 6.303; *P* = 0.003	Df = 3, 24; F = 0.586; *P* = 0.630	Df = 3, 24; F = 0.076; *P* = 0.972	Df = 3, 24; F = 1.992; *P* = 0.142	Df = 3, 24; F = 0.908; *P* = 0.530

The pH did not have an impact on the final larval or pupal weight. TFS and DFS had an effect on the final larval and pupal weights (*P* = 0.040). The larvae grown with DFS showed a final weight of 0.149 ± 0.0008g (Table 1).

The weight of the adult males was not affected by the pH of the diet or by the feeding system, while the weight of the adult females was significantly impacted by the pH of the diet: the females deriving from pH7.5 treatments differed from all other pH. The sex ratio, ingested food (%), and larva mortality (%) were only impacted by the feeding system, with the highest values reported for DFS. The sex ratio showed a significant interaction between the two factors as reported in Table 1. Percentage emergence was impacted by pH: pH7.5 showed differences from pH4.0 and pH6.1. The percentage of emergence was significantly higher (95.6 ± 2.8%) in DFS than in TFS (85.6 ± 5.8%). The time of development to 40% prepupae was not impacted by the pH of the diet, but was significantly different between feeding systems: the larvae provided with a single lump feeding (TFS) developed the faster (9.7 ± 0.24 d).

### pH evaluation

**Fig 3:**
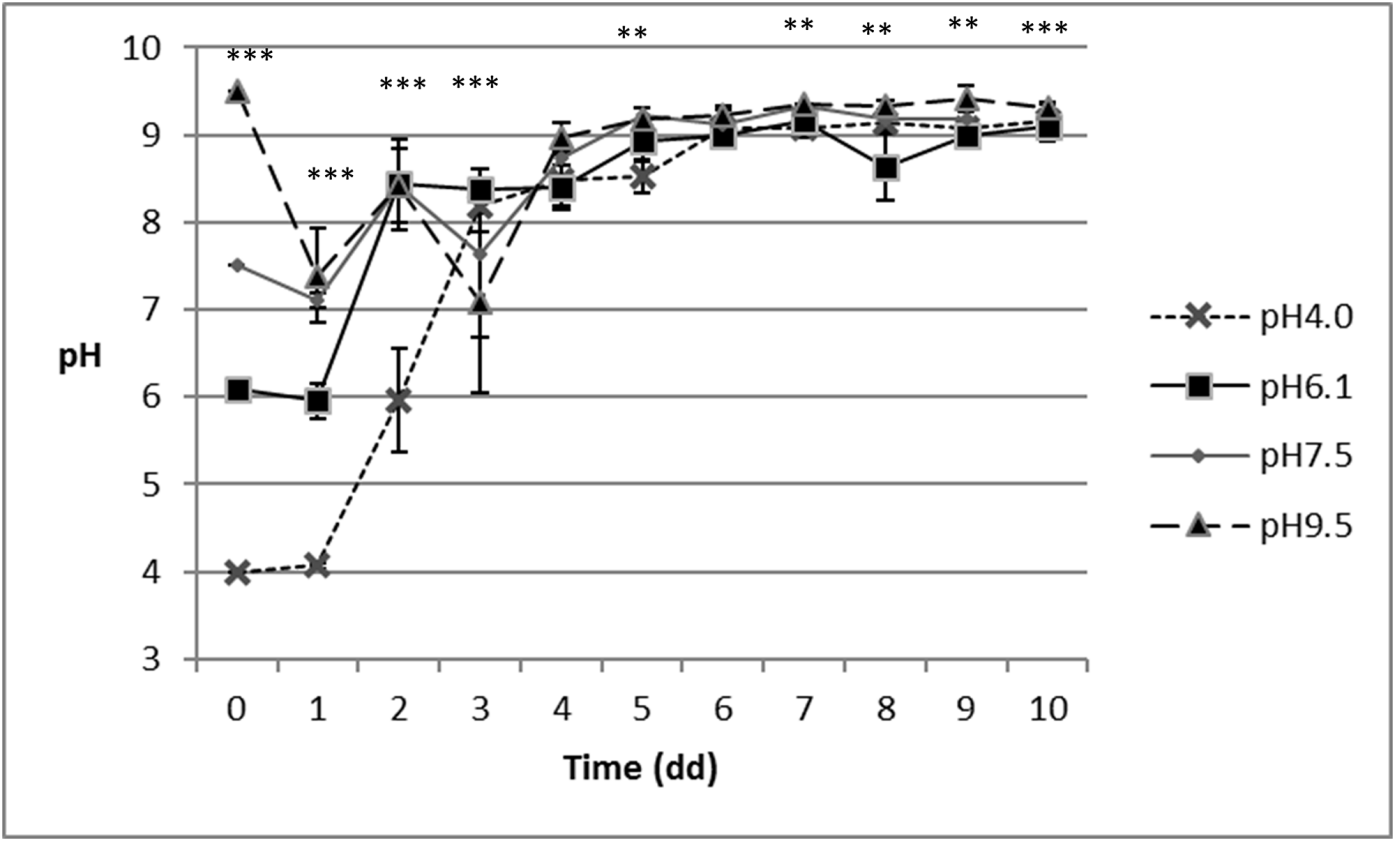
Changes in the pH over the course of the experiment, as a result of the feeding system and pH treatment. TFS: total amount of diet provided at the beginning (480g on WB).

**Fig 4:**
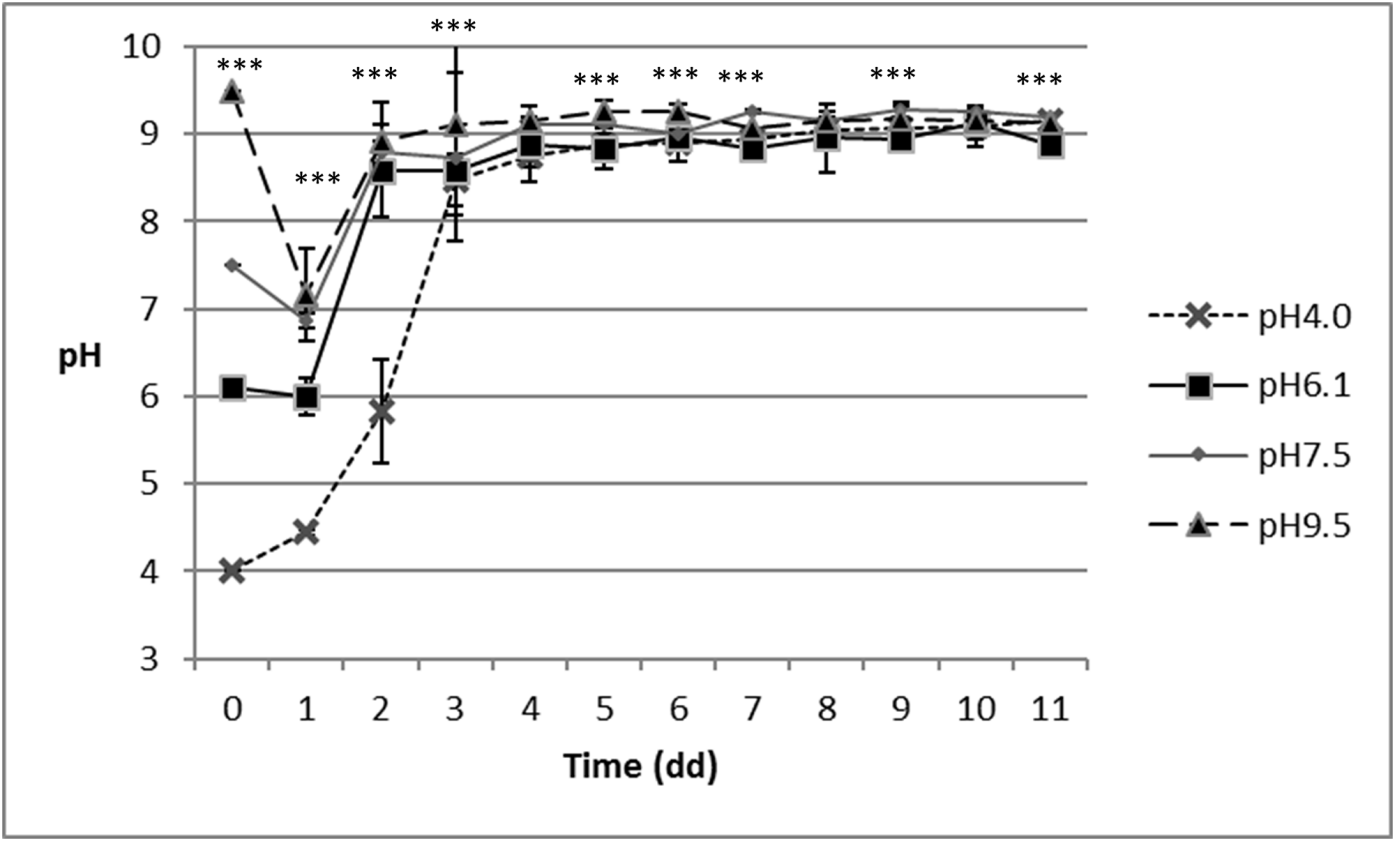
Changes in pH over the course of the experiment, as a result of the feeding system and pH treatment. DFS: daily allocated diet (40g/daily on WB).

Larval activity impacted the diet pH trend during growth, and the final values were around 9.0, as detected in more detail later on. No interaction between pH and feeding system was reported. The changes in the pH of the diet over time are presented in Figs 3 and 4.

Statistical analysis generally indicated differences between the pH treatments over the course of the experiment; the only days not showing any differences are the 4^th^ and 6^th^ days for TFS and the 4^th^, 8^th^ and 10^th^ days for DFS, as reported in Figs 3 and 4, respectively.

The results of a Post hoc comparison between the pH treatments from day 5 to end of the trial are reported in Table 2.

**Table 2:** Variation in the pH values (mean ± SD) from 5^Th^ day to end of the trial for the different initial pHs (pH4.0; pH6.1; pH7.5; pH9.5) and feeding systems (TFS: Total amount of diet provided at the beginning (480g on WB); DFS: Daily allocated diet (40g/d on WB)). Different letters within a column indicate significant difference (*P*≤0.05).

Feeding system	pH treatment	Day 5	Day 6	Day 7	Day 8	Day 9	Day 10	Day 11
**TFS**	**pH4.0**	8.5±0.19b	9.1±0.21	9.1±0.10b	9.1±0.12a	9.1±0.05b	9.1±0.06b	
	**pH6.1**	8.9±0.24a	9.0±0.11	9.2±0.16a	8.6±0.39b	9.0±0.07b	9.0±0.10b	
	**pH7.5**	9.2±0.10a	9.1±0.05	9.3±0.02a	9.2±0.11a	9.2±0.09ab		
	**pH9.5**	9.2±0.13a	9.2±0.10	9.3±0.01a	9.3±0.06a	9.4±0.15a	9.4±0.13a	
**DFS**	**pH4.0**	8.9±0.03c	8.9±0.04b	8.9±0.10ab	9.0±0.08	9.1±0.09a	9.1±0.18	9.2±0.11a
	**pH6.1**	8.8±0.04c	9.0±0.10b	8.8±0.10b	9.0±0.15	8.9±0.07b	9.1±0.05	8.9±0.13b
	**pH7.5**	9.1±0.05b	9.0±0.04b	9.3±0.11a	9.1±0.13	9.3±0.26a	9.3±0.05	9.2±0.00a
	**pH9.5**	9.3±0.08a	9.3±0.04a	9.1±0.26c	9.1±0.17	9.2±0.12a	9.2±0.17	9.1±0.08a

Regardless to the feeding system, the main changes in pH in all the treatments occurred within the first four days of the trial, after which, and till the end of the trial, pH values for TFS ranged between 8.5 (pH4.0) and 9.4 (pH9.5), and ranged from between 8.8 (pH6.1) and 9.3 (pH7.5) in DFS.

The final mean pH values of the two feeding systems were 9.2 ± 0.2 (TFS) and 9.1 ± 0.2 (DFS).

## Discussion

Our research demonstrated the feeding system impacted the growth performance of BSF larvae. Regardless of the feeding system, i.e. initial lump versus daily batches, BSF larvae are able to modify their feeding substrate within four day (Figs 3 and 4).

Moreover, our data demonstrate TFS resulted in the shortest development time, while DFS showed the heaviest final weight of the larvae. Moreover the differences in ingested food between TFS and DFS showed higher values in daily system compared to batch system. Considering batch system, it seems to be more efficient to gain time and to consumption of food in a mass rearing point of view. While daily system should be more efficient in a waste management treatment because larvae require more food and they consume it more than batch system.

These results could be important for realistic mass rearing productions with larger quantities of waste, if confirmed in such settings. However, a couple of parameters may be influenced differently e.g. by higher larval densities, which could result in a higher oxygen depletion and heat production than in such a pilot study as the present one [20]. Larva aeration and ventilation should also be considered in a mass rearing system, as they can be of major importance [20].

Ma et al. [38] evaluated a batch feeding system and examined the impact of the initial pH values of the substrate (pH = 2.0; pH = 4.0; pH = 6.0; pH = 7.0; pH = 8.0; pH = 10.0) on larval growth. They showed the heaviest weights were achieved for pH6.0 (0.21 g), for the control pH7.0 (0.20 g) and for pH10.0 (0.20 g), and there were no significant differences between them, while the lowest weight was found for pH2.0 (0.16g) and pH 4.0 (0.16g). Our results instead showed no differences in the final larva weight between the different pH treatments at the end of the trial. However, the feeding systems had an impact on the larval and pupal weights during the trial.

The male-to-female ratio was not influenced by the pH treatment. Sex rations were generally male-biased, a result in agreement with the results presented by Ma et al. [38] also reported a general higher number of males than females. Nevertheless, these authors reported a significant fluctuation of the ration as the pH changed, with the lowest value being recorded for the lowest pH. In contrast, we determined differences in sex ratio which potentially is a result of the interaction between pH and feeding system implemented, something not considered by Ma et al. [38].

All the final pH values of the diets after digestion ranged from 8.9 to 9.4. Similar pH values (8.9 and 9.0) were recorded in previous studies by Alattar [37] and by Popa and Green [36], respectively. Ma et al. [38] utilized the batch feeding system to evaluate modifications in the pH, and, differing from our results, they recorded a final acidic pH (6.0) of larvae reared on a diet with an initial pH of 4.0. The different results we found, compared to those of Ma et al. [38], could be ascribed to a different larva density and food distribution. In fact, in our research, the density was 60 larvae per liter, and a quantity of distributed food equal to 480 g per 500 larvae (0.96 g/larva), while in the trial by Ma et al. [38], the density was 100 larvae per liter for a food distribution of 500g for 300 larvae (≈1.6 g per larva). It is possible to hypothesize the change in pH could be correlated to the height of the provided food, which is influenced by the size of the container, by the density [37] and by the total amount of distributed food. Higher containers’ heights size have a different air circulation from that of lower ones, which could have an impact on fermentation at high temperatures (27–30°C). Unfortunately, Ma et al. [38] did not describe the size of their containers, and only the volume was mentioned. Our results have shown different kinds of feeding system could impact pH and larval growth. The larval weight differed between feeding systems from the second day of the trial onward. The TFS larvae had the heaviest weights, until the ninth day (Fig 2); however, those in the DFS treatment were the largest at the end of the experiment (Table 1, Fig 2). The cause of the heavier weights in the early days of the experiment for the TFS larvae could be ascribed to the delivery of the food altogether. This could influence the pH values [38] and the bacteria present in the diet during the trial, and the larvae could therefore have reached the optimum pH earlier than those fed under the DSF system. Moreover, the DFS larvae had to deal with a changing environment each day, which may have slowed down the feeding activity or caused a disturbance of the microflora. The TFS larvae grew faster than the DFS ones and required less time to reach the prepupa stadium than the DFs ones. Moreover, the daily delivery of the diet only had a negative impact on the growth of the DFS larvae in the early days of the experiment, as reported in Fig 2. However, the DFS larvae showed a higher final weight than the TFS larvae (Table 1). The co-digestion between larvae, bacteria and fungi may have reduced the food availability for the TFS larvae in the last days of the trial.

Our results showed BSF larvae are also capable of resisting and manipulating acidic (pH = 4.0) and basic (pH = 9.5) environments (as shown in Fig 3), thus confirming the findings of other researches with acidic pH. Alattar [37] demonstrated BSF larvae can survive in acidic environments (pH = 1.70), and Ma et al. [38] recorded a high survival, not only for an acidic diet (pH = 2, pH = 4 and pH = 6.0) but also for a neutral, pH = 7, and basic pH (pH = 8.0 and pH = 10.0). In our trial, mortality was not affected by the substrate pH, but only by the feeding system. Regardless of the pH of the substrate and of the feeding system, the here reported mortality was lower than those reported by Oonincx et al. [30] (ranging from 14 to 25%) and by Nguyen et al. [12], where mortality ranged between 19.2% and 52.8%. Conversely, our mortality results are slightly higher than the 2% of mortality reported by Tomberlin et al. [15].

Future research is needed to investigate factors impacting mortality rates. Doing so, could allow for optimal larval output.

Similar adaptations to pH have been observed in other families of Diptera. Larvae of the Yellow Fever mosquito, *Aedes aegypti*, L. (Diptera: Culicidae), and the black salt marsh mosquito, *Aedes* (*Ochlerotatus) taeniorhynchus*, (Wiedmann) (Diptera: Culicidae) have shown similar adaptations to low and high pH, respectively. A pH of between 4 and 11 did not negatively affect the growth of mosquito larvae or the hemolymph pH [43]. As reported by Chaudhury and Skoda [39], larvae of the blow fly, *C*. *hominivorax*, (Diptera: Calliphoridae), are able to survive on diets with pH 4.0 and pH 7.5; however, they determined larvae were smaller (51.6–53.8 mg) and had lower survivorship (47.8–59.9%) before reaching the pupal stage when reared in acidic, rather than basic diets. The larvae of the fruit fly, *C*. *capitata* Wiedemann (Diptera: Tephritidae), was found to survive over a wide pH range (3.2 to 8.0), with optimum growth occurring at a pH of 5.0–5.5 [40]. However, the mechanisms by which pH impacts the development of immature dipteran, and subsequently also their fitness, are currently not known.

pH could influence the availability of nutrients for BSF larvae by affecting performance of enzymes present in the gut of the larvae [44]. The enzymes of the salivary gland and of the gut of BSF larvae seem to play an important role in the ability of this species to digest different kinds of decaying organic matter [44]. The main discovered enzymes of the digestive apparatus are amylases, lipases and proteases. This enzymatic variability makes BSF a polyphagous species. The protease activity is in particular related to the pH, and the activity increases at a pH of 8.0, compared to lower acidic pH values. BSF larvae could operate a change in the substrate toward a basic pH value [44]. In our study, the BSF larvae alkalized the substrate up to 9.4. This could have promoted protease activity, which enhanced the protein availability, thus positively influencing invertebrate growth [45].

The abiotic conditions, such as pH, have an impact on the microbial communities within a given environment. Shifts in pH could also have an impact on other species, comprising the detritivorous community, and thus BSF. As reported by Lam et al. [46], house fly adults respond to the bacteria present in chicken manure. Similarly, house flies are highly selective regarding oviposition sites, especially if conspecific eggs are present. Lam et al. [47] determined bacteria proliferating on eggs changed over time. They determined if conspecific eggs were deposited prior to bacteria reaching a 10^6^–10^7^ CFU threshold in the oviposition substrate, the likelihood of survival increased significantly (more than 40% of larvae survived).

If the eggs were deposited after this threshold was reached, the larvae resulting from the already present eggs would cannibalize the deposited secondary eggs. These roles played by bacteria could be partially governed by the pH, which is known to partially regulate cell physiology, such as the anion and cation flows across cell membranes [48]. Similarly, the larvae of the fruit fly, *Dacus tryoni*, (Froggatt) (Diptera: Tephritidae), were not able to survive in the absence of certain bacteria, which were determined to be a part of their dietary needs [49]. This relationship between bacteria and larval fly development appears to be highly selective. Chandler et al. [50] demonstrated external and internal bacteria communities, associated with 14 species of *Drosophila* (Diptera: Drosophilidae), were highly restricted with four families, which represented 85% of the diversity.

Apart from regulating the oviposition site selection of the BSF [51], bacteria (e.g., *Bacillus subtilis*) have been used as a probiotic to enhance the larval development of this species [52]. In fact, the larval (0.0848–0.0946 g) and prepupal weight (0699–0.0766 g), as well as the adult body length (1.22–1.25 cm) increased, compared to specimens reared on a control diet (0.0775 g; 0.0606 g; 1.20–1.18cm; larvae, prepupae and adults, respectively) [52]. Thus, BSF larvae might modify the environment to a specific pH (8.5–9.2) in order to promote the growth of bacteria resulting in enhanced development and survivorship. Jeon et al. [53] showed the composition of bacteria communities in the gut of BSF larvae by pyrosequencing of the extracted intestinal metagenomic DNA, and they determined the composition was completely different from other species.

pH could also play a significant role on the growth and proliferation of pathogens, and could represent a food safety strategy [54]. *Escherichia coli* is sensitive to acidic environments [55] and values below 2.5 strongly inhibit its growth [56]. Furthermore, as reported by Mendonca et al. [57], high pH values (12.0) negatively affected Gram-negative bacteria (*E*. *coli* 0157:H7, *Salmonella enteritidis* ATCC 13706, and *Listeria monocytogenes* F5069), and destroy the microbial cell membranes.

In addition, BSF has shown the ability to reduce the populations of *S*. *enterica* and *E*. *coli* in chicken manure, by increasing the pH to 8.8–9.2. As previously reported by Erickson et al. [58], this could have been the result of the waxing level of ammonia, which is physiologically produced by the larval metabolism. The impact of ammonia on bacteria was also previously observed by Turnbull and Snoeyenbos [59]: increasing levels of ammonia in poultry manure progressively reduced the *S*. *enterica* population over a period of 11 days until it disappeared.

BFS is a polyphagous species that is able to grow under different substrate conditions. Our results demonstrated BSF larvae are able to survive over a wide range of pH, and larvae shift the pH to a more preferred pH condition, which, in this trial, seems to be around pH9. These findings confirm the results of previous studies and they help improve our understanding of this relationship and could be used to optimize the mass production of BSF under industrialized conditions. Special care should be taken to determine the pH of substrates provided to larvae in order to maximize the larval weight and production. Nevertheless, not only was the pH found to influence the development time, both of which were also influenced to a great extent by the feeding system. Even though no interaction was found in this research between these two main factors (pH and feeding system), further research is still needed concerning the relationship between pH and the associated microbial community within a substrate, as these factors most likely covary with and play an important role in regulating the growth and survival of BSF larvae.
